# A Case of Anti-glutamic Acid Decarboxylase-65 Antibody Positive Stiff Person Syndrome Presenting Initially as Acute Peripheral Vestibulopathy, Leading to Delayed Diagnosis After Multiple Hospitalizations

**DOI:** 10.7759/cureus.6083

**Published:** 2019-11-06

**Authors:** Yash Nene, Tejas Mehta, Sanjay Pawar, Gajanan Patil, Nasli R Ichaporia

**Affiliations:** 1 Neurology, Apollo Jehangir Hospital, Pune, IND; 2 Neurology, University of Missouri, Columbia, USA

**Keywords:** stiff person syndrome, vestibulopathy

## Abstract

Stiff person syndrome (SPS), with a prevalence of one to two per million, is an extremely rare neurological condition that is characterized by axial muscle stiffness and rigidity along with intermittent painful muscle spasms. It is often associated with psychiatric co-morbidities such as anxiety and depression. The pathophysiology, although poorly understood, is widely believed to be autoimmune in nature due to the association of anti-glutamic acid decarboxylase-65 (anti-GAD 65) antibodies with this condition. There is also a paraneoplastic variant that is more commonly associated with anti-ampiphysin antibodies. It occurs most commonly in patients with breast cancer followed by colon cancer. Most of the practising neurologists encounter just one or two cases of SPS in their entire careers, hence this condition remains underdiagnosed, leading to significant disability and distress to the patient. In this case report we describe a postmenopausal female who presented initially with symptoms of vertigo and dizziness and was hospitalized multiple times before the diagnosis was reached. Through this article, we attempt to increase awareness about this condition among practising physicians so as to increase the likelihood of earlier diagnosis and treatment.

## Introduction

First described by Woltman and Moersch in 1956 when they reported 14 patients, stiff person syndrome (SPS) is an extremely uncommon neuro-immunological condition characterized by axial muscle rigidity which is progressive in nature and stiffness along with episodes of painful muscle spasms [1]. Found to affect females more than males, it is categorized into four main variants -- classic, partial, paraneoplastic, and progressive encephalomyelitis with myoclonus and rigidity [2]. Treatment involves the use of GABAergic drugs such as benzodiazepines, along with corticosteroids, plasmapheresis, and intravenous immune globulin [2]. The pathophysiology is not completely understood, but is widely believed to be associated with the involvement of anti-glutamic acid decarboxylase (anti-GAD), which are cytoplasmic enzymes that take part in synthesizing GABA in the spinal cord and brain [3]. The GAD-65 isoform is associated with SPS, along with cerebellar ataxia, limbic encephalitis, and type 1 diabetes mellitus [4-6]. Approximately three out of five SPS patients are positive for anti-GAD antibodies [7], however, amphiphysin remains the dominant auto-antigen in paraneoplastic SPS [8]. Owing to the rarity of the condition, roughly 60% of cases are detected only because of the presence of anti-GAD-65 in the blood [9]. In this case report, we present an atypical case of SPS which, due to symptoms of vertigo and dizziness, was confused with peripheral vestibulopathy, leading to a delay in treatment.

## Case presentation

A 58-year-old previously healthy female patient presented to hospital with complaints of vertigo and imbalance for one week, along with back pain. She appeared anxious and her vital signs were within normal limits. Her neurological examination was essentially unremarkable except for bilateral gaze evoked nystagmus, more on the right side, and gait ataxia. Over the course of a four-day hospitalization, she underwent MRI of the brain and echocardiogram which were normal. While in hospital, she also had an episode of hyperventilation and blank staring look, which was attributed to panic episode at that time. A working diagnosis of acute peripheral vestibulopathy was made and she was treated symptomatically with betahistine and anxiolytics.

**Figure 1:**
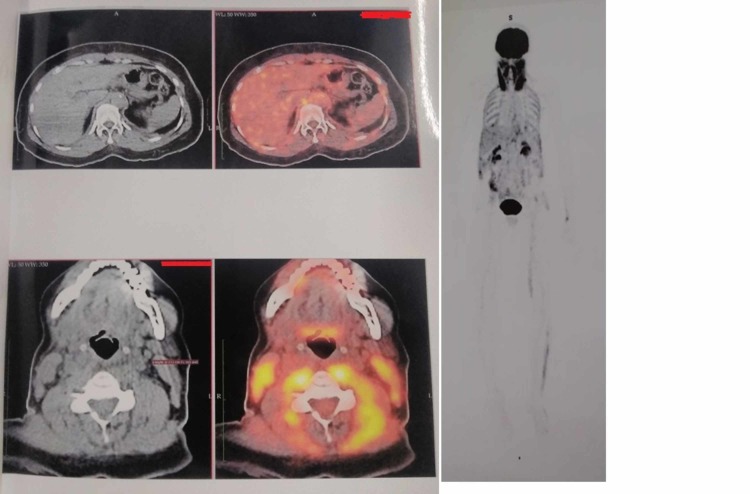
PET CT findings. PET: positron emission tomography

Her symptoms of vertigo and back pain along with nausea and headache persisted after being discharged. She also had blurry vision, gait ataxia, and neck stiffness. Twelve days after discharge, she was re-admitted to another hospital and investigated extensively. MRI brain and MRI spine were normal except for exaggerated lumbar lordosis. Cerebrospinal fluid (CSF) analysis revealed glucose of 70 mg/dL (normal 45-80 mg/dL), protein of 34 mg/dL (normal 15-60 mg/dL), and 5-8 cells/mL (normal 0-5 cells/mL) with 94% lymphocytes and 6% neutrophils. Hemogram and basic metabolic panel were normal. Due to possibility of paraneoplastic syndrome, whole body positron emission tomography (PET) scan was done which revealed diffusely increased 18-fluorodeoxyglucose (FDG) uptake in sternocleidomastoid, pre-vertebral as well as muscles of thoracoabdominal wall suggestive of myositis (Figure 1). 

Creatine phosphokinase (CPK) was found to be 695 (normal 26-192 U/L). She was treated symptomatically with analgesics, anti-emetics, anti-depressants, gabapentin, and propranolol over a one-week hospitalization. At this stage, the diagnosis was still unclear but a few conditions like subacute cerebellar ataxia, paraneoplastic syndrome, and essential oil toxicity were being considered.

She was brought to us a week after this episode. Her back pain and difficulty walking had persisted. She also had diffuse headache with giddiness along with back spasms and left leg weakness. On examination, she was wheelchair bound, conscious, and co-operative. She was unable to stand and ambulate without assistance and had severe tenderness in her neck and back muscles, along with tightness and stiffness in the trapezius. On examination, her deep tendon reflexes were brisk and power was 4/5 in all four limbs. Sensations were intact and cerebellar function was normal. Notably, she was always keeping her left leg in a flexed position and complained of pain on moving the left hip. She was admitted again to be investigated. Whole body MRI revealed marrow edema within left acetabulum and right femoral head showing heterogenous postcontrast enhancement with surrounding enhancing soft tissue suggestive of neoplastic etiology, concerning for metastasis/marrow infiltrative disorder. Subsequent bone biopsy was largely normal, revealing only scanty tissue showing bony trabeculae. Anti-nuclear antibody (ANA) and urine Bence Jones protein were negative. CPK level was 262. Anti-glutamic acid decarboxylase (GAD 65) antibodies were markedly elevated at >2000I U/mL (normal <10 IU/mL). This along with the constellation of characteristic symptoms established the diagnosis of SPS. Treatment with intravenous immune globulin (IVIG), diazepam, baclofen, steroids, and mycophenolate were started. She complained of intractable vomiting on the second day of treatment, so all oral feeds were stopped. Esophagogastroduodenoscopy revealed large hiatal hernia and gastric ulcers, which were treated accordingly. Post this, IVIG was restarted. Her symptoms improved dramatically, with progressive increase in muscle strength and mobility and decrease in hip pain and spasticity. She was soon able to ambulate without support and was discharged on oral medications. 

## Discussion

Stiff person syndrome’s uncommon occurrence and its elusive signs make the diagnosis of this condition difficult. Patients generally present insidiously, with episodic stiffness and pain of the axial musculature gradually progressing to the proximal muscles. Hyperlordosis of the lumbar spine is pathognomonic [10]. This case report demonstrates how, due to the rarity of the condition, the diagnosis is often overlooked, leading to delayed diagnosis and treatment. Our patient also initially presented with atypical cerebellar symptoms instead of the classical pain and stiffness, leading to multiple hospitalizations and extensive workup before the diagnosis was made. 

A very typical but often underappreciated feature of this disease is marked anxiety, excessive startle, and specific phobias with the latter being a rather common feature as compared to the others mentioned [11]. The symptoms of ataxia and vertigo, and anxiety coupled with the hyperventilation and staring spells made the diagnosis a challenge during the first visit, leading to missed or delayed diagnosis of SPS. This delay in diagnosis led to deterioration of the patient’s condition, repetitive hospital admission, and loss of precious time and resources.

Our case report highlights the need for practicing neurologists to have a reasonably high index of suspicion for diagnosing SPS, a rare but progressively debilitating autoimmune disease of the central nervous system, which is easily treatable but often overlooked.

## Conclusions

This case demonstrates how considerable time and resources were spent and the patient continued to be in distress before the correct diagnosis was reached. SPS may not always present with the classical stiffness and rigidity and may present with atypical features such as gait and balance problems, blurry vision, and headache. Features such as exaggerated startle reflex and hyperlordosis of the lumbar spine point strongly to the diagnosis. Treating neurologists must always have this possibility in consideration when they encounter a patient with neurological signs and symptoms that are difficult to localize and cannot be explained adequately by an infectious/inflammatory/neoplastic process. Assay for GAD-65 antibodies can provide a prompt diagnosis, thereby reducing patient discomfort and saving time and resources. If clinical suspicion remains high, benzodiazepines, anti-spastic medications and intravenous immune globulin can be started even before results of GAD-65 antibody testing are available. 
